# Endoscopic Submucosal Dissection for Esophageal Adenocarcinoma Amidst Variceal Vulnerability

**DOI:** 10.7759/cureus.106671

**Published:** 2026-04-08

**Authors:** Fred Ahmadi, Saif Affas, Roberto Gamarra

**Affiliations:** 1 Internal Medicine, Ascension Providence Hospital, Southfield, USA; 2 Gastroenterology, Ascension Providence Hospital, Southfield, USA

**Keywords:** barrett’s esophagus, endoscopic submucosal dissection, endoscopy, esophageal adenocarcinoma, esophageal varices

## Abstract

Endoscopic submucosal dissection (ESD) has become a curative therapy for early esophageal adenocarcinoma (EAC), yet the presence of esophageal varices poses significant challenges. We present the case of a 67-year-old man with alcoholic cirrhosis and varices who was found to have a short-segment Barrett’s esophagus containing a mass at the gastroesophageal junction. Subsequent biopsy confirmed intramucosal adenocarcinoma of the esophagus. Given the patient's poor surgical candidacy, ESD was performed. This case illustrates that carefully planned ESD can be safely executed in select patients with EAC and esophageal varices when standard therapy is contraindicated. It also highlights the possibility of high-risk malignancy even in short-segment Barrett’s esophagus.

## Introduction

Esophageal adenocarcinoma (EAC) is a common malignancy associated with Barrett’s esophagus and chronic gastroesophageal (GE) reflux disease. For superficial lesions, endoscopic mucosal resection (EMR) and endoscopic submucosal dissection (ESD) are preferred minimally invasive alternatives to esophagectomy, preserving organ function and reducing morbidity [1]. However, the presence of esophageal varices due to portal hypertension introduces a high risk of catastrophic bleeding that often precludes these procedures. Data on ESD in this context remain limited to a handful of reports and studies [2,3].

Traditional management of early EAC in patients with varices has relied on systemic or palliative approaches because surgery is often contraindicated. This case highlights two considerations: (1) the technical possibility of ESD for early EAC in the presence of esophageal varices and (2) the occurrence of high-risk malignancy within even short-segment Barrett’s esophagus.

## Case presentation

**Figure 1:**
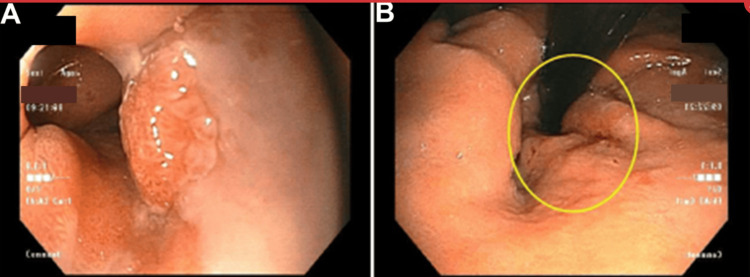
Esophagogastroduodenoscopy revealing a mass at the gastroesophageal junction (A), which is extending into the gastric cardia (B, circle).

A 67-year-old Caucasian man with a history of alcoholic cirrhosis and tobacco use underwent esophagogastroduodenoscopy (EGD) for the evaluation of Barrett’s esophagus. Initial EGD revealed mucosal changes consistent with Barrett’s at approximately 1 cm in length and a medium-sized, non-obstructing, fungating mass at the gastroesophageal (GE) junction extending into the stomach cardia, with grade II esophageal varices beneath the lesion (Figure 1).

**Figure 2:**
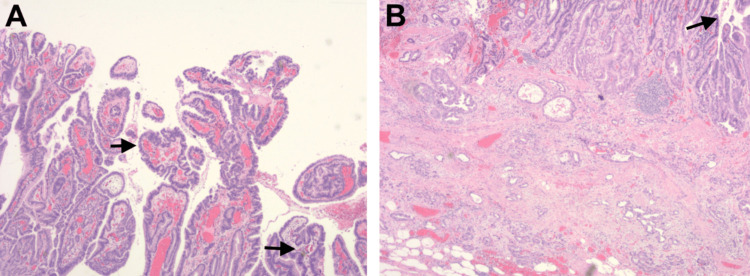
Biopsy showing villous epithelial proliferation (A) and the base of the lesion with invasive glands extending into the submucosa (B).

Biopsy of the lesion showed small nests of neoplastic cells within the lamina propria, consistent with intramucosal adenocarcinoma (Figure 2).

**Figure 3:**
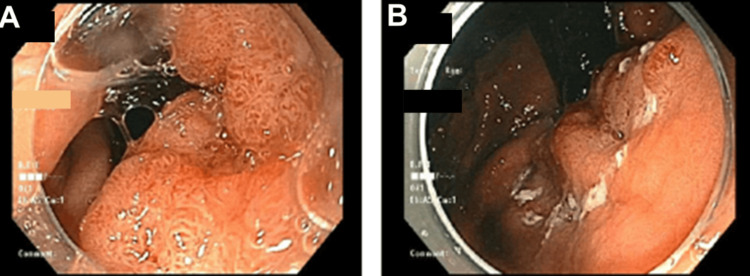
Repeat esophagogastroduodenoscopy revealing a mass that has increased in size at the gastroesophageal junction (A) and extending into the gastric cardia (B).

One month later, repeat EGD demonstrated a friable, partially circumferential mass at the GE junction (Figure 3).

Varices were again noted in the lower and mid-esophagus, although difficult to visualize in the figure.

**Figure 4:**
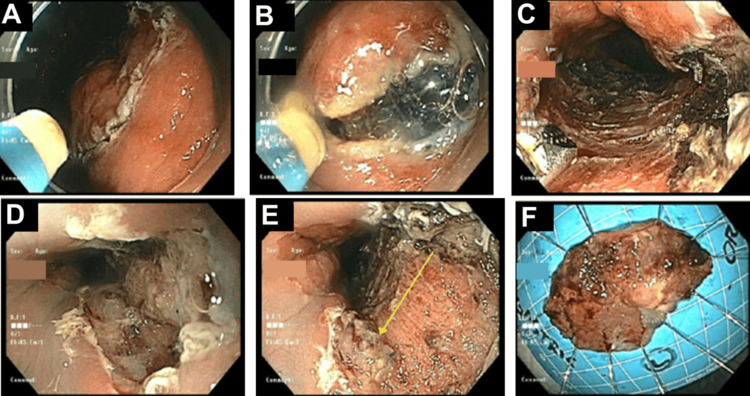
Esophagogastroduodenoscopy revealing a circumferential cut around the lesion, which is created by a dual knife (A), followed by lifting of the lesion from the muscularis propria (B). This is followed by dissection of the lesion from the underlying layers (C, D) and separation of the lesion entirely (E, arrow.) The lesion was retrieved with a Roth net and is seen here (F).

Given the patient’s underlying cirrhosis and poor surgical candidacy, a multidisciplinary decision was made to proceed with ESD. Thermal marking was performed to outline lesion borders and achieve clear demarcation. Methylene blue solution was injected into the submucosa to create adequate lift and delineate the plane from the muscularis propria. A dual knife was used to make a circumferential incision around the lesion (Figures 4A, 4B), followed by careful dissection of the submucosal layer (Figures 4C-4E) using electrocautery. The resected specimen measured approximately 3 × 5 cm, representing 50% of the circumference of the esophagus and gastric cardia along the posterior wall. The lesion was retrieved with a Roth net (Figure 4F).

All visible vessels were pre-coagulated to minimize the risk of delayed bleeding, and hemostasis was achieved without the use of band ligation or sclerotherapy. No intra- or post-procedural bleeding occurred. Resection margins were negative for tumor involvement. The patient recovered without complication and was discharged on a proton-pump inhibitor with scheduled endoscopic follow-up. A follow-up CT at the patient’s next office visit was negative for any recurrence of disease or metastasis.

## Discussion

Our case highlights several important issues, first being the possibility of ESD in a patient with EAC and concomitant esophageal varices and the importance of recognizing malignant potential even in short‐segment Barrett’s esophagus.

First, the curative role of ESD in early EAC is now well‐established in selected patients without high-risk features (e.g., deep submucosal invasion, lymphovascular invasion) and who are poor surgical candidates. Endoscopic therapy (including ESD) offers organ preservation, lower morbidity, and shorter recovery compared with esophagectomy. However, the presence of esophageal varices adds a layer of complexity. Varices carry a risk of major bleeding, both intra‐procedurally and post‐procedurally, and many centers consider varices a relative contraindication to endoscopic resection.

In a multicenter Japanese study of 1,708 patients undergoing ESD for superficial esophageal cancer, 27 (1.6%) had esophageal varices. Among that group, en bloc resection was achieved in 100% and R0 in 77.8%. Importantly, there was no statistically significant increase in adverse events compared to patients without varices [3]. Similarly, a 2022 case report described successful treatment of early esophageal squamous cell carcinoma overlying varices without bleeding complications after prior variceal therapy [2]. These data show that while varices pose a risk, they do not necessarily preclude endoscopic intervention.

Our case also shows the concept that even short‐segment Barrett’s esophagus (in this case ~1 cm) can give rise to high‐risk lesions. While surveillance guidelines [4] stratify risk by length of Barrett’s segment, nodular or mass‐forming lesions, even in short segments, warrant high suspicion and prompt biopsy and staging. From a pathology standpoint, the lesion in our case measured ~3 × 5 cm and involved the lower esophagus and gastric cardia with negative margins, again highlighting the value of en bloc resection with ESD. En bloc resection is one of the major advantages of ESD compared to EMR and allows more precise risk stratification and avoidance of under-treatment [1].

## Conclusions

This case illustrates that early EAC can arise even within short-segment Barrett’s esophagus, reinforcing the need for vigilance during surveillance endoscopy regardless of segment length. It also demonstrates that the presence of esophageal varices, while often considered prohibitive, does not preclude curative therapy in appropriately selected patients. As experience and evidence continue to grow, ESD may expand the therapeutic options. Future prospective studies are needed to determine further uses of ESD as well as long-term outcomes of these patients.
